# The Superstitions Connected with the History and Practice of Medicine and Surgery

**Published:** 1844-10-01

**Authors:** 


The Superstitions connected with the History and Practice of Medicine and Surgery. By *Thomas Joseph Pettigrew,* F.R.S. F.S.A. &c. &c. &c. 8vo. pp. 167.

In this utilitarian age the literature of the profession runs a risk of being confined to weekly Journals, manuals for students, practical compendiums for medical readers, and popular or quasi-popular works for the public. Theory is not to the taste of the day, which has outgrown it—discovery is becoming something like an impossibility—transcendentalism and German mysticism itself has got a bottom, which we *must* touch before long—the microscope has magnified till it can magnify no longer—and chemistry, with its seemingly inexhaustible crucible, seems the only store likely to hold out.

However, with all our "practical" tastes, we have many of us time enough (and something more) to turn over the leaves of such a book as this, and smile or sneer, as our humour may be, at the follies of those who have gone before us. Buried follies no doubt—the credulities that wait on ignorance, and such as are scarcely likely to revive. But civilization has its superstitions, science has its mysteries and its fanaticisms too, the human mind is not purged, and we dare say it never will be, of that love for authority, confidence in bold assertion, and liking for the marvellous, which have made men in all times, the tools and dupes of quacks in physic, religion, and politics. In this enlightened nation, priding itself upon its arts, and arms, and all that springs from civilization and refinement, the machinery for taking the reason prisoner, for enslaving the mind to dominate the body, for quackery, in short, and humbug, is in as active operation, though more subtly worked, as in a Byzantine palace or a Papal Court. The actors are changed, the dresses are different, the piece is cut down from a play to a farce, but the plot is similar, and the denouement is the same.

No doubt there are those who entertain a more consolatory and more flattering opinion of the progress of the human mind. They talk of the enlightenment of the age, and really believe that it is superior to bigotry and prejudice. Perhaps they cite, in support of their view, the miracles of Hoenlohe, Irving, and the Mesmerists, the cures of Morrison, and the wonders of Homœopathy and of Hydropathy.

For our own parts we are less sanguine. The mass of men will, we fear, be enslaved by names, and the willing dupes of their own inclinations and of others' arts. They have, at all events, no great right, at present, to laugh at the credulousness of their fathers.

Mr. Pettigrew's book is amusing to such as are inclined to smile, and instructive to those who are willing to reflect. The reader should sit down to its perusal, with the motto—" mutato nomine de te," &c. before him. To read it thus will do him service, and he will shut up the volume, a less arrogant, if if not a wiser, man than when he opened it.

The "Introduction," with which the work commences, is intended to illustrate the text—that "man is a dupeable animal," and a sort of natural prey for quacks of all descriptions. The first really great quack in medicine was Paracelsus, and a quack of the first water he certainly was.

"He was professor of medicine at Basle, but became renowned by a nostrum called *azoth*, which he vaunted as the philosopher's stone—the medical panacea —the tincture of life. He styled himself the 'monarch of physicians,' and arrogantly explained that the hair on the back of his head knew more than all authors; that the clasps of his shoes were more learned than Galen or Avicenna; and that his beard possessed more experience than all the academy of Basle: 'Stultissimus pilus occipitis mei plus scit, quam omnes vestri doctores, et calceorum meorum annuli doctiores sunt quam vester Galenus et Avicenna, barba mea plus experta est quam vestræ omnes Academiæ.' Extravagant as all this may appear, it yet had the effect of dissipating a too excessive admiration of the ancients, at that time prevalent in the schools. His boldness was such, that at his first lecture upon his appointment to the professorship in the University, he, before his pupils, publicly burnt the writings of Galen and Avicenna! His education, however, was very imperfect, and he was ignorant even of his own vernacular tongue. Thomas Erastus, one of his pupils, wrote a book to detect his impostures. He was nevertheless a man of great ability, and did much towards the advancement of chemical knowledge, particularly in its application to the purposes of medicine. Armed with opium, antimony and mercury, he effected many extraordinary cures." 4.

There are few quackeries or quacks that have not some truth in the midst of all their lies, though the kernel is frequently small enough. Still quackery does a little good, if it does, at the same time, a great deal of harm. It rouses attention, prevents apathy and a torpid acquiescence in what is established, on the part of the profession, and sometimes gives a hint which may be followed out with benefit. The per contra, however, are heavy, and carry down the balance fearfully. The great evil of quackery is the stimulus that it gives to the credulity of men. An evil of incalculable magnitude, and one that would outweigh, a thousand times, all the good that quackery ever did. Then look at the falsehoods it breeds. Born in it, nurtured by it, what a system of lying does it not engender. It is impossible to assign limits to the damage which such profligate mendacity must inflict upon the public morals.

A Chapter on Alchymy naturally enough succeeds the Introduction. So soon as men had any natural science, the pursuit of wealth and the prolongation of life were likely enough subjects to occupy them. The transmutation of metals and the philosopher's stone were the foundation of chemistry. This occult art has beeen attributed to the Arabians, and Geber mentions the philosopher's stone. So little did Dr. Johnson appreciate the science of this philosopher, that he disrespectfully supposes that the word gibberish has been derived from the peculiar style of his writings. Mr. Pettigrew, however, renders it not improbable, if he does not actually prove, that the Egyptians have a good right to the invention of Alchymy.

"Alchymy," says he, "cannot be regarded as of Arabian origin, however much it may have been cultivated and extended in that country. It flourished at a very early period in Egypt, and the late discoveries in that 'land of marvels' have shown an extended acquaintance with various arts and sciences as exercised in the different manufactures, of which representations are to be found in the tombs and excavations of a very early date. Without some knowledge of chemistry the Egyptians could never have excelled, as they have done, in the making of glass, of linen, in dyeing, in the use of mordaunts, &c. Their manufacture of metals, particularly of gold—the whole process of which is represented in the tombs of Beni Hassan and at Thebes—into various ornaments; their gold wire, their gilding, &c. exhibit great ability, and could not have been effected without some knowledge of metallurgy. Their embalmings also display an acquaintance with chemistry. The Egyptian manuscripts hitherto discovered have not afforded any particular light into the extent of their knowledge; but several papyri have been found to contain certain formula; and one, a bilingual manuscript (being Enchorial and Greek) was examined by my late friend, Professor Reuvens, the conservator of the Museum of Antiquities at Leyden, and was found to treat of magical operations, and to contain upwards of one hundred chemical and alchymical formulæ.

"It has been usual to ascribe the introduction of alchymy to Pythagoras, to Solomon, or rather to Hermes, and it has not unfrequently been called the Hermetical science. Gibbon has shown that the Greeks were inattentive either to the use or the abuse of chemistry, and that the immense collection of Pliny contains no instance of, or reference to, the transmutation of metals. He states the persecution of Diocletian to be the first authentic event in the history of alchymy. After the conquest of Egypt by the Arabs it spread over the globe." 7.

That, in the infancy of chemistry, there should be alchymists is not wonderful. The compositions and decompositions of the metals, &c. are wonderful enough at the present day, when we fairly understand them. They who believed in this transmutation, cannot be looked on as extraordinary dupes. It was not an unnatural idea. Every body knows that men of all ranks, and of great accomplishments *did* believe. Perhaps the most extraordinary public *act* of belief was that of Henry VI.

"Henry IV. had enacted a statute prohibiting the craft of multiplication. None were permitted to multiply gold or silver, under pain of felony. Henry VI. repealed this statute and published a patent *authoritate Parliamenti,* which has been given by Prynne in his 'Aurum Reginæ,' and in which the monarch tells his subjects that the happy hour was drawing nigh when, by the discovery of the philosopher's stone, he should be enabled to pay all the debts of the nation in real gold and silver." 9.

We are afraid His Majesty's lieges never saw the royal *tin*, much less the gold.

Elias Ashmole actually received, in *syllables*, from his adopted father, Backhouse, a wretched astrologer, who lived a beggar notwithstanding, " the true matter of the philosopher's stone. Of this he professes that he knew enough to hold his tongue, but not enough to speak." And yet, in the most approved nosological fashion, he laid down four varieties of the stone in question—to wit, the mineral stone—the vegetable stone—the magical stone—and the angelical stone. Lest any should be disposed to doubt, or smile, he solemnly tells us that " incredulity is given to the world as a punishment," a doctrine that has always found favour with those who had got a faith to establish. However, the properties of the stones in question ought not by any means to be unknown :—

" The *mineral stone* hath the power of transmuting any imperfect earthy matter into its utmost degree of perfection; that is, to convert the basest of metals into perfect gold and silver; flints into all manner of precious stones, as rubies, sapphires, emeralds, diamonds, &c.

" The *vegetable stone*, by which Abraham, Moses, and Solomon wrought many wonders. The nature of man, beasts, fowls, fishes, all kinds of trees, plants, flowers, &c. may by this stone be made to grow, flourish, and bear fruit,—increase in colour, smell, &c. when and where and at whatever season of the year its possessor may please.

" The *magical or perspective stone* makes a strict inquisition, discovers any person in any part of the world whatever, and enables you to understand the language of birds, beasts, &c.

" The *angelicall stone* can neither be felt, seen, or weighed, but it can be tasted. It will lodge in the fire to eternity without being prejudiced. It hath a divine power, celestial and invisible, and endows the possessor with divine gifts. It affords the apparition of angels, and gives a power of conversing with them by dreams and revelations, nor dare any evil spirit approach the place where it is." 10.

Some may, perhaps, ask—" why rake up the rubbish of such buried balderdash." Buried *it* may be, but imposture as errant, humbug as gross, courts its dupes and its abettors in the present day. With what face can that age laugh at Alchymy which tolerates the Mesmerists. The latter gentry seem to be of the Alchymist's way of thinking, and act as though they had got old Chaucer's rhyme by heart :—

" Make privy to your dealing as few as you maie,
For three may keepe councell if twain be awaie."

The belief in the transmutation of metals has not yet died out in the minds of chemists. Dr. Christopher Girtanner, an eminent professor of Göttingen, has recently prophesied :—

" In the nineteenth century the transmutation of metals will be generally known and practised. Every chemist and every artist will make gold; kitchen utensils will be of silver, and even gold, which will contribute more than anything else to prolong life, poisoned at present by the oxyds of copper, lead, and iron, which we daily swallow with our food." 15.

In the language of John Gilpin—

" May we be there to see."

After Alchymy, we are presented with a chapter on Astrology. It is hard to say which was the greater folly of the two, though, perhaps, astrology will, without difficulty, carry off the palm. The latter is the pure offspring of ignorance and fear, the other has some connexion with science. It would be difficult to tell what good astrology has done the world, unless, as was the case with Newton, it may be looked upon as having led to the cultivation of astronomy. But alchymy has been the foundation of that great lever of human progress—Chemistry.

It is not very flattering to medicine, that astrology was considered indispensable to the accomplished physician, and to physicians of the old school, very probably it was. The wisdom of *our* ancestors was great in medical matters.

"Red flowers were given for disorders of the sanguiferous system, yellow ones for those of the biliary secretion, &c. We find that in smallpox red bed-coverings were employed, with the view of bringing the pustules to the surface of the body. The bed-furniture and hangings were very commonly of a red colour,—red substances were to be looked upon by the patient. Burnt purple, pomegranate seeds, mulberries, or other red ingredients were dissolved in their drink. In short, as Avicenna contended that red bodies moved the blood, everything of a red colour was employed in these cases. John of Gaddesden, physician to Edward II., directs his patients to be wrapped up in scarlet dresses: and he says that 'when the son of the renowned king of England (Edw. II.) lay sick of the smallpox, I took care that everything around the bed should be of a red colour; which succeeded so completely that the Prince was restored to perfect health, without a vestige of a pustule remaining.' Wraxall, in his 'Memoirs,' says that the Emperor Francis I., when infected with the smallpox, was rolled up in a scarlet cloth, by order of his physician, so late as 1765, when he died. Kæmpfer (History of Japan) says that 'when any of the emperor's children are attacked with the smallpox, not only the chamber and bed are covered with red hangings, but all persons who approach the sick prince must be clad in scarlet gowns.' Flannel died nine times in blue was held to be efficacious in the removal of glandular swellings.

"The rising and setting of the stars, the eclipses of the sun and moon, the appearance of comets or other fiery meteors, the aspects, conjunctions, and oppositions of the planets, have all been considered to be intimately influential in the production as in the relief of diseases. Fracastorius, a poet and physician, sought for the causes of diseases in the heavens. Certain positions of the celestial bodies he considered to be of malignant influence, by which contagious disorders were produced. A conjunction of many stars under the large fixed stars predicted a contagion, falling stars and comets denoted putrefaction. The Jesuit Kircher, after a strict examination of almanacks and astrological tables, contended that putrid diseases had always prevailed at those times when the planets Mars and Saturn were in conjunction. He therefore inferred that those two planets emitted very deadly exhalations, which infected the air and all terrestrial productions with a putrescent tendency,—when myriads of animalcules were instantly generated, and the plague, the smallpox, the measles, or some other putrid fevers, became inevitable.

Mr. Fraser* notices the superstitious belief of the natives of Khorasān in the powers of the celestial bodies, and says they attributed the ravages of the cholera with which they were visited to the influence of the star Canopus, (called by the Persians and Arabians Zoheil,) which became visible at this time above the horizon, a little before sunrise." 20.

---

* Narrative of a Journey into Khorasan, p. 64.

We may laugh at John of Gaddesden, but his reasoning was just as good as some of our own. It was after all but the popular "post hoc ergo propter hoc" argument that he resorted to. Your experimenters of the present day do, often, nothing else. And we should like to know what can be more scientific than Kircher's way of doing business. Were he alive now, he would be one of the most active members of the Statistical Society. Nothing can be more à-la-mode than his reasoning. He looks over the old almanacks, (history, we know, is nothing else,) and finds when putrid diseases prevailed—he consults the astrological tables, and ascertains that just then Mars and Saturn were always in conjunction. What can be more satisfactory—what more statistical—what more conclusive. We are sure that the statistical people can neither pursue a more satisfactory method, nor often arrive at a more instructive result.

Nor is a belief in the influence of the planets altogether a tradition. In the "clime of the sun," not only the vulgar, but the faculty, too, believe in Lunar and Sol-lunar influence.

> "Dr. Balfour, who for a long time resided at Calcutta, an accurate and intelligent observer of the diseases which occur in hot climates, is generally considered to have satisfactorily established the influence of the moon in cases of fever, and he was induced, during a practice of fourteen years in the East, to pay particular attention to its revolutions in the treatment of these diseases. He found the accession of fever to take place during the three days which either precede or follow the full moon; and he has endeavoured to show that, at the time of the equinoxes, an additional power is added to the lunar influence exercised on the human frame. These opinions have met with support and received confirmation from the practice and researches of Lind in Bengal, of Cleghorn in Minorca, of Fontana in Italy, of Jackson in Jamaica, of Gillespie at St. Lucia, of Bell in Persia, and of Annesley in Madras. Dr. Moseley carried his opinions with respect to the influence of the moon on mankind to a ridiculous extreme, and affirmed that almost all people of extreme age died at the new or at the full moon. Aristotle ascribes many of the derangements of females to the decrease of the moon. Galen says all animals born when the moon is falciform are weak and feeble, and short lived; whilst those born at the full are the contrary. Lord Bacon invariably fell into a syncope during a lunar eclipse." 21.

If the moon influences the tides, why may it not influence us? If the sun has such an effect, as it unquestionably has, on the health and the organization of animals and plants, why may not the moon have some? It would be very shabby to deny it any. However, not being *lunatics*, we shall waive the question, and refer it to Mr. Farre, who, we dare say, will take it up in his next Report. With the following, we shall dismiss the subject of astrology.

> "In savage nations the physicians, if they may be so called, are all conjurors and wizards, persons supposed to be gifted either with divine or demoniacal natures. Incantations, sorcery, jugglery of all kinds, engrafted, probably, in many cases upon enthusiasm, together with ignorance, supply the place of science, to which they are utter strangers. Whatever is beyond their sagacity is assigned to invisible agency. Pliny calls magic the offspring of medicine, and says that, after having fortified itself with the help of astrology, it borrowed all its splendour and authority from religion." 24.

It is certainly paying no compliment to religion, and not a great one to physic, to find that when the latter is lowest it is in alliance with religion, while in its present palmy state the world (we believe, however, unjustly) accuse it bitterly of scepticism. It must be recollected that when physic was humbug, religion was superstition, and the reformation of the latter has left the science of the former to its future path and course. Whatever that may be, it must be owned, at all events, that in these days we are, with the exception of the Mesmerists, &c., no conjurors. But really what *were* Early Medicine and Surgery.

If Bacon is to be believed, it was no better than it should be.—"Witches and impostors," he says, "have always held a competition with physicians," implying, we presume, that they were a good deal alike. However that may be, Le Clerc contends that medicine came immediately from God, and Adam is allowed by the Fathers of Medicine to have been the first general practitioner. No surgical instruments have been discovered among the implements found in the tombs of Egypt, and so soon as the physician and surgeon were become separate individuals, the former took precedence, by reason of the skill necessary for the detection of internal diseases, and we doubt also of the little skill exhibited in the treatment of external ones. How could it be otherwise, and what sort of surgeons must they have been who knew nothing of anatomy!

It is odd, perhaps, that circumcision should be the first surgical operation on record. It was a holy rite, and executed by the priests. The first book on anatomy was written by a sovereign, Athotis, the first king of Egypt, founder of Memphis. Many of our readers, we dare say, are acquainted with Herodotus' account of the state of Egyptian physic and physicians. "Every physician was for one disease—not more; so that every place was full of physicians; for some were doctors for the eyes, others for the head; some for the teeth, others for the belly; and some for occult disorders. There were also physicians for female disorders. The sons followed the profession of their fathers, so that their number must necessarily have been very great. Herodotus says παντα δε ιητρων επιπλεα. This arrangement and classification, however, could scarcely have taken place until the practice of medicine had ceased with the priesthood." Practically a high state of civilization—our present one, at all events, leads to something of the same sort. We have our liver-doctors, stomach-doctors, water-doctors, &c., and, while the public approve of the arrangement, there will be plenty in the profession to encourage the taste for it. In fact, if we all start with a good education, if we are all on a level with the science of the day, and there is no mystery in the matter, there is perhaps no great harm in one man's devoting himself much to one class of complaints, another man to another class.

The Egyptians, however, went rather farther than we do. They divided the human body into thirty-six parts, each of which they believed to be under the particular government of one of the decans or aerial demons, who presided over the triple divisions of the twelve signs; and we have the authority of Origen for saying, that when any part of the body was diseased, a cure was effected by invoking the demon to whose province it belonged." It naturally followed that the planets should come to exercise dominion on the several parts of our mortal frames, as may be learnt from that work of useful knowledge—the almanac of the immortal Francis Moore, physician. Southey has drawn up a sketch of this zodiacal system, that may possibly amuse, if not instruct.

"There Homo stands, naked but not ashamed, upon the two Pisces, one foot upon each; the fish being neither in air nor water, nor upon earth, but self-suspended as it appears in the void. Aries has alighted with two feet on Homo's head, and has sent a shaft through the forehead into his brain. Taurus has quietly seated himself across his neck. The Gemini are riding astride a little below his right shoulder. The whole trunk is laid open, as if part of the old accursed punishment for high treason had been performed upon him. The Lion occupies the thorax as his proper domain, and the Crab is in possession of the abdomen. Sagittarius, volant in the void, has just let fly an arrow, which is on the way to his right arm. Capricornus breathes out a visible influence that penetrates both knees; Aquarius inflicts similar punctures upon both legs. Virgo fishes as it were at his intestines; Libra at the part affected by schoolmasters in their anger; and Scorpio takes the wickedest aim of all." 31.

The history of medicine in connection with the church is not a little curious, though far from creditable to the clergy. Physic being of heavenly origin, the priests laid hold of it and monopolised it. We may naturally suppose what it was in their hands—a thing of omens, prayers, divination, and every species of imposture. To such an extent did this go, that it became a sacerdotal means of swindling the sick and dying to such a tune, that Councils and Popes were obliged, for very shame, to interdict the shepherd from shearing the flock so close, and to forbid the priesthood from visiting the sick otherwise than as ministers of religion. For some time the attempts to arrest the rapacity of the holy men was fruitless, and the result is instructive.

"When the priests ascertained that they could no longer confine the practice of medicine to themselves it was stigmatized and denounced. At the Council of Tours, held in 1163 by Pope Alexander III., it was maintained that the devil, to seduce the priesthood from the duties of the altar, involved them in mundane occupations, which, under the plea of humanity, exposed them to constant and perilous temptations. They were accordingly prohibited the study of medicine, and that of the law, and every ecclesiastic who should infringe the decree was threatened with excommunication. In 1215 Pope Innocent III. fulminated an anathema specially directed against surgery, by ordaining, that as the church abhorred all cruel or sanguinary practices, no priest should be permitted to follow surgery, or to perform any operations in which either instruments of steel or fire were employed; and that they should refuse their benediction to all those who professed and pursued it.

"Stringent as these measures were, they were found inadequate to effect the purpose intended, and it was only at length accomplished by a special bull procured from the Pope, which, by permitting physicians to marry, effectually divorced medicine from theology." 34.

A pretty piece of history. But, after all, the Romish Church has by no means given up its trade in physic. A snug business is done in shrines, relics, and saints. The latter respectable fraternity have taken a leaf out of the book of Egyptian medicine, and adopted most successfully the division of labour. The following edifying list will be useful to all true believers. It comprises the more prominent patrons of a few of our complaints.

"St. Agatha, against sore breasts.
St. Agnan and St. Tignan, against scald head.
St. Anthony, against inflammations.
St. Apollonia, against toothach.

St. Avertin, against lunacy.
St. Benedict, against the stone, and also for poisons.
St. Blaise, against the quinsy, bones sticking in the throat, &c.
St. Christopher and St. Mark, against sudden death.
St. Clara, against sore eyes.
St. Erasmus, against the cholic.
St. Eutrope, against dropsy.
St. Genow and St. Maur, against the gout.
St. Germanus, against the diseases of children.
St. Giles and St. Hyacinth, against sterility.
St. Herbert, against hydrophobia.
St. Job and St. Fiage, against syphilis.
St. John, against epilepsy and poison.
St. Lawrence, against diseases of the back and shoulders.
St. Liberius against the stone and fistula.
St. Maine, against the scab.
St. Margaret and St. Edine, against danger in parturition.
St. Martin, against the itch.
St. Marus, against palsy and convulsions.
St. Otilia and St. Juliana, against sore eyes and the headach.
St. Pernel, against the ague.
St. Petronillai, St. Apollonia, and St. Lucy, against the toothach.
——————, and St. Genevieve, against fevers.
St. Phaire, against hæmorrhoids.
St. Quintan, against coughs.
St. Rochus and St. Sebastian, against the plague.
St. Romanus, against demoniacal possession.
St. Ruffin, against madness.
St. Sigismund, against fevers and agues.
St. Valentin, against epilepsy.
St. Venise, against chlorosis.
St. Vitus, against madness and poisons.
St. Wallia and St. Wallery, against the stone.
St. Wolfgang, against lameness." 38.

It is well to know to whom to apply in case of our doctor being out of the way. Certainly some of the Saints are condescending. No less than four look after toothache—three might be supposed enough to render lithotrity unnecessary. St. Blaise might have been invoked with advantage, if not success, by Mr. Brunel, while St. Maine, for the scab, St. Martin for the itch, and St. Job and St. Fiage for the p—x, are, to say the least, more civil than particular.

There has always been a sovereign virtue in wells and fountains. No doubt, if people confined themselves to drinking what flows from them, there would be less intemperance and fewer headaches than are actually going. But the sacred fount has its tutelar divinity, and the Naiad still lives in the less euphonious persons of St. Quan, St. Brogawn, et hoc genus omne of saints. The well of the two last-named gentlemen is a sample of the whole. Mr. and Mrs. Hall inform us that: —

"The times for visiting it are the last three Sundays in June, when the people imagine the saints exert their sacred influence more particularly for the benefit of those who apply for their assistance. It is confidently said, and firmly believed, that at this period the two saints appear in the well in the shape of two small fishes of the trout kind; and if they do not so appear, that no cures will take place. The penitents attending on these occasions ascend the hill barefoot, kneel by the stream and repeat a number of paters and aves, then enter it, go through the stream three times, at a slow pace, repeating their prayers. They then go on the gravel-walk, and traverse it round three times on their bare knees, often till the blood starts in the operation, repeat their prayers, then traverse three times round a tree on their bare knees, but upon the grass. Having performed these exercises, they cut off locks of their hair and tie them on the branches of the tree as specifics against headach. The tree is a great object of veneration, and presents a curious spectacle, being covered all over with human hair." 41.

We are not sure that there is not something in this. If bilious folks would practice the gymnastics and the water-cure enjoined by St. Quan and Co. regularly, the probabilities are that their attacks would be few.

A TALISMAN is defined by Mr. Pettigrew to be a substance composed of certain cabalistical characters engraved on stone, metal, or other material, or else written on slips of paper. It differs from an amulet in this respect, that it may be deposited in any place, or carried about the person without losing its efficacy, whilst the latter requires to be constantly worn about the individual.

But Fosbrooke has, with his usual and his useful accuracy, shewn us that there are really five sorts of Talismans, a thing worthy to be known, as we can choose to our tastes or necessities. There are, then :—1. The *Astronomical*, with celestial signs and intelligible characters. 2. The *Magical*, with extraordinary figures, superstitious words, and names of unknown angels. 3. The *Mixed*, of celestial signs and barbarous words, but not superstitious or with names of angels. 4. The *Sigilla Planetarum*, composed of Hebrew numeral letters, used by astrologers and fortune-tellers. 5. *Hebrew Names and Characters*. These were formed according to the cabalistic art, and the whole form a complete and valuable collection. A writer in the Gentleman's Magazine has been good enough to translate for the world in general and his readers in particular, a Hebrew Talisman, we presume of the fifth sort. Nothing can be more calculated to inspire both confidence and veneration than the charm.—It reads :— "It overflowed—he did cast darts—Shadai is all sufficient—his hand is strong, and is the preserver of my life in all its variations." It appears that there are talismanic characters on all the old houses still standing in Edinburgh. Can the Kirk hear this unmoved ?

An AMULET means, in Arabic, "that which is suspended," and the Greeks and Romans set us the example of using them pretty extensively. In the legal "Duel," the champions were obliged to take an oath, "that they had ne charm ne herbe of virtue," a fair and necessary precaution, consistent with the whole of that sensible specimen of the wisdom of our ancestors.

"In its composition the amulet is of the most varied kinds; objects selected either from the animal, the vegetable, or the mineral kingdoms; pieces of old rags or garments, scraps of writing in legible or illegible characters, in fact, of anything to which any superstitious property has been considered to belong. Sir Walter Scott had in his possession one of these pretended charms taken from an old woman who was said to charm and injure her neighbours' cattle. It consisted of feathers, parings of nails, hair, and such like trash, wrapped in a lump of clay." 48.

It is pleasing to find that amulets are thus within the reach of all classes, and our fair friends may have as good an one out of their old flannel petticoats, as royalty itself. There was a great trade carried on in African amulets, called *grigris*, which amongst other desirable properties, had the anti-Malthusian one of procuring the fortunate possessor many wives, and giving to them easy deliveries. No wonder that the trade in *grigris* has fallen off. Heaven forbid it should ever revive, at all events, in this country.

Sentences, substances such as camphor, precious stones have all been used as amulets. "The necklaces so commonly found on the Egyptian mummies are usually composed of objects having a funereal or mythological character or import, and were doubtless used as amulets to preserve the integrity of the body—a circumstance peculiarly essential with the Egyptians, being in accordance with their system of theology." The chrysolite is an amulet worth having. *Inducit sapientiam fugat stultitiam.* Unfortunately, as every body knows, chrysolites are rare. We cannot sufficiently praise the sound discretion of the Mischna, which permits the Jews to wear amulets, provided they have been found efficacious in at least three cases, by an approved person.

Odd numbers are notoriously serious matters. "Some philosophers are of opinion that all things are composed of number, prefer the odd before the other, and attribute to it a great efficacy and perfection, especially in matters of physick: wherefore it is that many doctors prescribe always an odd pill, an odd draught, or drop to be taken by their patients. For the perfection thereof they allege these following numbers: as 7 Planets, 7 Wonders of the World, 9 Muses, 3 Graces, God is 3 in 1, &c." Very conclusive reasons. It is evident that the belief is rather general in the profession, many doctors, and of name too, writing *very odd* prescriptions, and doing equally odd things. No doubt Dr. Elliotson has a *numeral* amulet.

While on this subject let us not forget to report, in due course, the medical virtues inherent in an odd sort of person—the seventh son of a seventh son. He is born a physician, knowing diseases intuitively, and often curing by the touch only. And why should he not, when Priessnitz, Morrison, and the rest do as much, or more, without any pretension to being born in the least unlike their neighbours. We are sorry however to be obliged to state, that M. Thiers' reasons point rather unfavourably than otherwise for seventh sons:—"Plusieurs croyent qu'en France, les septièmes garçons, nez de legitimes mariages, sans que la suitte des sept ait, esté interrompue par la naissance d'aucune fille, peuvent aussi guerir des fièvres tierces, des fièvres quartes, et mesme des ecrouelles, après avoire jeûné trois ou neuf jours avant que de toucher les malades. Mais ils font trop de fond sur le nombre septenaire, en attribuant au septième garçon, preferablement à tous autres, une puissance qu'il y a autant de raison d'attribuer au sixième ou au huitième, sur le nombre de trois, et sur celuy de neuf, pour ne pas s'engager dans la superstition. Joint que de trois que je connois de ces septièmes garçons il y en a deux qui ne guerissent de rien, et que le troisième m'a avoué de bonne foy, qu'il avoit en autrefois la reputation de guerir de quantité des maux, quoique en effet il n'ait jamais guery d'aucun. C'est pourquoy Monsieur du Laurent a grande raison de rejetter ce pretendu pouvoir, et de la mettre au rang des fables, en ce qui concerne la guerison des ecrouelles."

It is hard enough that, in France, eldest sons should not have the advantage of their primo-geniture, without cutting off from seventh sons their perquisites too. M. Thiers is very much to be blamed.

The two magical words ABRAXAS and ABRACADABRA have had a world of commentaries written on them, and played a great part in their day. The latter fearful sign was recommended by Serenus Samonicus, a physician in Caracalla's reign, as an amulet to cure ague, and other diseases. The letters comprising Abracadabra are to be so written, that reading from the apex on the right and up the left side, the same word will be given as at the top:

ABRACADABRA
BRACADABR
RACADAB
ACADA
CAD
A

or thus:

ABRACADABRA
ABRACADABR
ABRACADAB
ABRACADA
ABRACAD
ABRACA
ABRAC
ABRA
ABR
AB
A

Julius Africanus says that pronouncing the word is as good as writing it. Julius Africanus is no doubt right.

CHARMS are of much the same kidney as Amulets, only the latter were to be suspended when employed, which the former were not necessarily to be. Charms being derived from carmen, were frequently verses in which the spells were written. Cato the Censor found the reduction of a dislocation readily effected by singing IN ALIO S. F. MOTAS VAETA, DARIES DARDARIES ASTATARIES DISSUNAPITUR. That capital surgeon, however, had other strings to his bow, for if the dislocation did not yield to the above chaunt, he had two still better:—HUAT HANAT ISTA PISTA SISTA, DOMIABO DAMNAUSTRA, et luxato. Vel hoc modo, HUAT HAUT ISTA SIS TAR SIS ARDAUNABON DAMNAUSTRA." No dislocation, we apprehend, *could* resist this.

That scraps of verse, pieces of old rag, coral, beads, and pins and needles should keep people well and render them invulnerable, cannot surprise any body. As we are fond of historical evidence, we may cite Marco Polo, in attestation of the fact. He writes " that, in an attempt by Kublai Khan to make a conquest of the island of Zipangu, a jealousy arose between the two commanders of the expedition, which led to an order for putting the whole of the inhabitants of the garrison to the sword; and that, in obedience thereto, the heads of all were cut off, excepting of eight persons, who by the efficacy of a diabolical charm, consisting of a jewel or amulet introduced into the right arm, between the skin and the flesh, were rendered secure from the effects of iron, either to kill or wound. Upon this discovery being made, they were beaten with a heavy wooden club, and presently died." A good instance this of the way in which the devil *sells* his friends. For if they had had no charm at all they would simply have had their heads cut off—but being defended by Satan, all they got was a good deal of hacking and hewing to no purpose (the heads being fixtures), and a beating to death after all.

It is worth observing, as Mr. Pettigrew does, that the principal complaints for which charms were efficacious, were those which are much under the influence of the nervous system, the imagination, and so forth. Epilepsy, convulsions, and fits have been the staple for which charms were in request. It is curious with what instinct, hypocrisy and enthusiasm have always acted. Down to this very day, epilepsy is the quack's favourite. It is on epilepsy that Mesmerism lays its hands, and that and hysteria have been its great guns all along. It is not because these complaints are so apt to go suddenly of themselves, or from some strong impression of the mind, that they have been selected by the quacks ; no, it is simply because they are frightful and intractable maladies.

It is not wonderful that charms should be resorted to against the plague, that terrible disorder, sudden in its onset, frightful in its course, defying human skill, and looking like a direct infliction of divine vengeance. The notions of the Moors on the subject may be interesting now, when that people have come into collision with Europe, and are likely enough to come under the sway of France at no very distant period.

" Mr. Jackson, consul at Mogodor, in his 'Travels in Africa,' gives us an account of the plague which depopulated West Barbary in 1799-1800, and says that the Mahomedans, who are predestinarians and believe in the existence of spirits, devils, &c., regard the plague as a good or blessing sent from God to clear the world of a superfluous population ; that no medicine or precaution can cure or prevent it ; that every one who is to be a victim to it is (*mktube*) recorded in the book of fate ; that there are certain genii who preside over the fate of men, and who sometimes discover themselves in various forms, having often legs similar to those of fowls ; that these genii are armed with arrows ; that when a person is attacked by the plague, which is called in Arabic *l'amer*, or the destiny or decree, he is shot by one of these genii, and the sensation of the invisible wound is similar to that from a musket-ball ; hence the universal application of *M'drab* to a person afflicted with the plague, i. e. he is shot, and if he die *ufah ameruh*, his destiny is completed or terminated—in the world. Mr. Jackson says he scarcely ever yet saw the Mooselmin who did not affirm that he had, at some time of his life, seen these genii, and they often, they say, appear in rivers.

" Fear, he observes, had an extraordinary effect in disposing the body to receive the infection ; and those who were subject thereto invariably caught the malady, which was for the most part fatal." 66.

It is not a little in favour of the value of human testimony, that these Mooselmin affirm that they have *seen* these genii. It would be difficult to find a lie, however palpable, that witnesses could not be got, or have not been got, to swear to. As for credulity, the experience of our own day shews that *it* is not confined to the uneducated. It is rife in all ranks, and it ever was so. The powder of toad was, like many other things, employed as a preservative against the plague. "Pope Adrian is reported never to have been without it. The ingredients forming his amulet were dried toad, arsenic, tormentil, pearl, coral, hyacinth, smarag, and tragacanth. Among the Harleian MSS. is a letter from Lord Chancellor Hatton to Sir Thomas Smith, written at the time of an alarming epidemic. He writes thus: 'I am likewise bold to recommend my most humble duty to our dear mistress (Queen Elizabeth) by this LETTER and RING, which hath the virtue to expell infectious airs, and is *to be worn betwixt the sweet duggs,* the chaste nest of pure constancy. I trust, sir, when the virtue is known, it shall not be refused for the value.'" Whether the Virgin Queen did actually wear the said ring "betwixt the sweet duggs," deponent sayeth not, and history is silent.

We have latterly cultivated morbid anatomy with some success, but it is questionable whether we have not gone back in therapeutics. The worst of it is, that the modern race of medical men are so indifferently read in the olden literature of their profession. Many valuable facts are thus buried in unopened volumes, to the great loss of the public, and the disgrace of themselves. We shall resuscitate a hint or two for the benefit of the present age.

In *ague,* we are many of us insufficiently aware of the value of the chips of a gallows, put into a bag, and worn round the neck. Yet a little consideration might point out the probability of that application being a valuable one; for the gallows itself must have effectually cured the ague of the gentleman who swung on it.

That spiders cure ague is a fact too incontestably established to admit, we presume, of any doubt. Elias Ashmole's evidence would be, alone, conclusive. "I took early in the morning a good dose of elixir, and hung three spiders about my neck, and they drove my ague away. Deo Gratias!" More pious, however, than logically satisfactory; for the sceptical might remark, that possibly the elixir drove away the ague, or possibly it went away of itself, nay, perhaps, it did not go at all. But there are great authorities in favour of the spiders:—Diascorides, Matthiolus, Aldrovandus, and a host of old women.

We need not go into the instructive case of the Hon. Robert Boyle, who cured his ague by applying to his *wrists* a mixture of bay salt, hops, and blue currants; nor will we, with Mr. Boyle, explain how the remedy *does* cure. But we cannot refrain, for the benefit of our friends in the Fens, from mentioning another remedy or two, which cannot fail to give satisfaction.

"In Skippon's account of a 'Journey through the Low Countries,' &c., he makes mention of the lectures of Ferrarius and his narrative of the cure of the ague of a Spanish lieutenant, by writing the words FEBRA FUGE, and cutting off a letter from the paper every day, and he observed the distemper to abate accordingly; when he cut the letter F last of all the ague left him. In the same year, he says, fifty more were reported to be cured in the same manner.

"Another charm for ague was directed to be said up the chimney, by the eldest female of the family, on St. Agnus Eve. It ran thus:—

"Tremble and go!
First day shiver and burn:

Tremble and quake!
  Second day shiver and learn:
Tremble and die!
  Third day never return."

Some cunning people go at dead of night five different times to the nearest cross-road, and there bury a new-laid egg. Of course, the ague's in it. But they take good care not to speak to anybody, for that would break the charm, if not the egg. Other persons, of equal sense, break a salted cake of bran, and give it to a dog, when the fit is coming on. The dog naturally gets, and the patient loses it. Serenus Samonicus has put us in possession of as agreeable, and, we dare say, as effectual a remedy as any. It is placing under the head of one with *quartan ague* the fourth book of Homer's Iliad—we imagine in *quarto*.

In *Hectic Fever and Consumption*, the canny Scotchmen "pare the nails of the fingers and toes of the patient, put them into a rag cut from his clothes, then wave their hand with the rag thrice round his head, crying, *Deas soil;* after which they bury the rag in some unknown place."

We would strongly advise any one who has a child ill with the hooping-cough, to look out for some one on a *pye-ball* horse, and ask him for a remedy for the *hooping-cough*. Whatever he names is an infallible specific.

That scrofula is the "king's evil" every body knows; that it was cured by the king's touch most, we fancy, will admit. But not only would the king's touch do it—that of a virgin (not easily got), and of a murderer (what a compliment to majesty), would do as well.

The treatment of rickets is ingenious, and illustrates the popular phrase of being "in a cleft stick." We were not, however, aware of the advantages derivable from that position.

"Grose says that if a tree of any kind is split, and weak, rickety, or ruptured children drawn through it and the tree afterwards bound up, as the tree heals and grows together, so will the children acquire strength. Sir John Cullum saw the operation performed, and states that the ash tree was selected as the most preferable for the purpose. It was split longitudinally about five feet: the fissure was kept open by the gardener, whilst the friend of the child, having first stripped him naked, passed him thrice through it, almost head foremost. This accomplished, the tree was bound up with packthread, and as the bark healed, so it was said the child would recover. One of the cases was of rickets, the other a rupture. The ash tree has been very generally preferred for superstitious practices. White, in his well-known 'Natural History of Selborne,' has noticed this particularly. Not only trees, but stones, were similarly used. To creep through a perforated stone was a Druidical ceremony; and in the parish of Marden there is, or was, a stone with a hole in it fourteen inches in diameter, through which children were drawn for the rickets." 75.

We all know that Erysipelas is *St. Anthony's Fire*, but our particular obligations to the Saint are not, perhaps, so familiar as they ought to be. It appears, then, that erysipelas was once raging violently throughout Europe, and that, thanks to St. Anthony, it miraculously ceased. It is a pity that, whilst he was about it, he did not put a stop to it for good.

If our readers have any fair friends with warts, we are sure they will thank them for the following valuable recipe.

"Grose gives, for the removal of these excrescences, direction to 'steal a piece of beef from a butcher's shop, and rub your wart with it, then throw it down the necessary-house, or bury it, and as the beef rots, your warts will decay.' Sir Thomas Browne says, 'for warts we rub our hands before the moon, and commit any maculated part to the touch of the dead.'

Sir Kenelm Digby says, 'One would think it were a folly that one should offer to wash his hands in a well-polished silver bason, wherein there is not a drop of water, yet this may be done by the reflection of the moonbeams only, which will afford it a competent humidity to do it; but they who have tried it, have found their hands, after they are wiped, to be much moister than usually; but this is an infallible way to take away warts from the hands, if it be often used.'" 80.

"Oh! Sir Kenelm Digby," unbelievers will protest, "what a liar art thou! Ferdinand Mendez Pinto was but a type of thee!" and yet we dare to say that Sir Kenelm did wash his hands with moonbeams, the washing being a matter of moonshine. And it is highly probable that the hands of some of our acquaintance, which to the vulgar eye seem merely dirty, undergo a similar ablution.

The history of the remedies for hæmorrhage might be read with advantage by most people now. Look at the styptics advertised—look at the belief entertained by eminent men, in the virtues of Ruspini's, and think, if you will, the present age altogether superior to the past. True they relished the marvellous. "Boetius de Boot says he cured a maid at Prague, who had suffered from a violent hæmorrhagy for six years, for which she had often been bled, and various remedies resorted to without effect, by merely hanging a jasper round her neck which effected her cure. Upon leaving off the jasper the hæmorrhage would return, and this continued to be the case for some time; at length, however, she was perfectly cured.

"Van Helmont affirms that he had a metal, of which, if a ring were made and worn, not only the pain attendant upon hæmorrhoids would cease, but that in twenty-four hours, whether internal or external, they would vanish altogether."

Very precise evidence, and given on the personal testimony of two respectable men. As for charms, it would be useless to quote them. Almost any saint in the calendar would answer the purpose. We cannot, however, resist the opportunity of once more introducing the celebrated Robert Boyle, the chemist and natural philosopher. He says:—"Having been one summer frequently subject to bleed at the nose, and reduced to employ several remedies to check that distemper; that which I found the most effectual to stanch the blood was some moss of a dead man's skull, (sent for a present out of Ireland, where it is far less rare than in most other countries,) though it did but touch my skin, till the herb was a little warmed by it."

There are those who, ever deferring to authority, would silence all doubts by the reply that, "it was believed by this or that eminent man." But such is the weakness, such the inconsistency of the human intellect, that however it may shine in one direction that is no guarantee against lamentable blindness in another. The case of Boyle is one of the many in point.

It might be expected that there would be many superstitions connected with an event so interesting to the human race, as child-birth. There are more indeed than we have a mind to chronicle. One or two however, are amusing.

"The men in some countries, when the women are delivered, lie in, keep their bed, and are attended as if really sick. At Surinam, Fermin says, the man keeps to his *hamac* for six weeks. In Persia, when a woman is about to lie in, the schoolmasters are requested to give liberty to their boys, and birds confined in cages are permitted to escape. Charlevoix says that when the women of Maroc perceive labour-pains, the neighbours select five school-boys, and tie four eggs in the four corners of a napkin, with which the boys run singing through the streets." 86.

We every now and then see advertisements in the papers, to the effect that a *child's caul* is to be sold. Perhaps some of our readers do not exactly know what a child's caul is.

"In this and some other countries when a child is born with the caul or amnion over its face, it is preserved with great care and regarded as ominous of good fortune to the infant, and also as valuable to any one who may become possessed of it, enabling them to avoid many serious dangers. 'Il est né coiffé,' is a French proverb applied to lucky people. In Scotland, according to Ruddiman, it is called a *haly* or *sely how*, a holy or fortunate cap or hood. A midwife in Scotland is called a howdy or howdy wife. The virtues of the caul are described as various; it renders advocates eloquent, saves the possessor from having his house destroyed by fire, or being himself drowned. It is therefore much in request with sea-faring people." 86.

No doubt they must find their account in it, or they would not pay so much as they do for it.

The INFLUENCE OF THE MIND UPON THE BODY is a teeming subject, and might occupy volumes as easily as chapters. It is a phenomenon which we all experience in ourselves, observe continually in others, reflect upon not much, and act upon less than we might. The routine of practice comes to make us regard blue-pill, or senna, bark, or iodine as our efficient weapons against disease, and partly from our so regarding them, partly from the mind and its armoury of influences not being so readily at our disposal, we are too apt to neglect the latter, and to resign them in all their powerful applications to the quacks and impostors who use them with unprincipled, and often with successful recklessness.

It would not be amiss, as Dr. Abercrombie has suggested, if the philosophy of the mind were made a branch of medical education far more than it is. If any one ought to understand it, a physician should, for it is an integral part of physiology, and its observation is as integral a part of his professional life. Yet how few in the profession are really acquainted with it.

If we cite some instances of the effects of the passions and sentiments upon the body, it is not because medical men are ignorant of their existence, but because they are in themselves of interest as specimens.

That *Fear* has caused death is very well known, and *Joy* has had its victims. "Sophocles, at an advanced age, and in the full possession of his intellectual power, composed a tragedy, which was crowned with such success that he died through joy. Chilon of Lacedemon died from joy whilst embracing his son, who had borne away the prize at the Olympic

Games. Juventius Thalma, to whom a triumph was decreed for subjugating Corsica, fell down dead at the foot of the altar at which he was offering up his thanksgiving. Fouquet, upon receiving the intelligence of Louis XIV. having restored him to liberty, fell down dead."

Rather a droll instance of the effects of apprehension was submitted to the Royal Academy of Sciences at Paris, in 1823.

"M. Buisson had attended a woman in hydrophobia; he bled her: his hands were smeared with the blood, and he wiped them with a cloth which had been applied to remove the saliva from the mouth of the patient. M. Buisson had an ulceration upon one of his fingers at the time, which circumstance no doubt dwelt upon his mind. On the ninth day after his attendance he was suddenly seized with a pain in his throat and eyes, the saliva was continually discharging itself from his mouth, the impression of a current of air, the appearance of a shining substance occasioned him painful sensations, his body seemed to him so light that he felt as though he could leap to a prodigious height, and he had a desire to bite—not men, but animals and inanimate bodies. He now began to drink with difficulty, and the sight of water was exceedingly distressing to him. He traced the pain of his throat, which recurred every five minutes, as extending from his finger up the arm to the shoulder and neck. He conceived himself labouring under hydrophobia; and horribly impressed by its fatality, he resolved to terminate his existence by stifling himself in a vapour-bath. He caused the temperature of the bath to be raised to 42°, (107°·36″ Fahr.) when he was equally surprised and delighted to find himself completely well. He left the bathing-room, dined heartily, drank more than usual, and remained perfectly free from all complaint." 102.

Unquestionably M. Buisson, labouring under hydrophobia, must have been endowed with more than Roman valour, when he resolved to die in *a vapour bath.* This was devoting himself, in Curtian fashion to the infernal gods. Happily the whole ended as it did, in smoke, or what is nearly the same thing, steam. The denouement, a dinner, and a bottle of wine is agreeable.

The phenomena of Catalepsy and Hysteria are amongst the most curious, and, practically to us as medical men the most important of those, which evince the mutual action of the mind and body. On the one hand, we have the *will* in operation, and a resolute and clever cheat shows us how far the usual manifestations of bodily suffering can be endured without seeming consciousness. On the other, the sentiments, passions, imagination predominate, and the hysterical girl or the nervous enthusiast, present the same phenomena of extravagant emotion or endurance. Impostors and visionaries of this description have gulled the world in every past age, and probably will gull it in every future one. The mass of men think little, reason less on facts beyond the range of their common experience. They see what is to them unusual, what appears miraculous, and they believe it what it seems. With the public credulity we cannot quarrel. To expect philosophy from those untrained to philosophical investigations would be unreasonable and absurd. But what must move us to laughter or to spleen, as our humour is wont to look on the infirmity of our nature, is when men of education and intelligence are as grossly humbugged as the meanest of the mob. This it is which makes us despair of the destiny of our race, and doubt if the æra will ever arrive when it will cease to be led by the nose.

But to return to our author. He alludes to those convulsive actions produced by imitation which are among the most singular of the sort.

"Mr. Cornish, of Falmouth, gave a curious account of several instances in which he observed the effects of this mental excitement. It took its rise in a Wesleyan chapel in Redruth, and extended to others of the same denomination in Camborne, Helston, Truro, Penryn, and Falmouth. During the time of divine service, a man called out loudly and unexpectedly, 'What shall I do to be saved?' and expressed a most alarming apprehension as to the state of his soul. The example thus set was speedily followed, and the individual affected seemed to suffer great bodily pain. This circumstance becoming known, hundreds flocked to the chapel from curiosity, and many became similarly affected. The chapel was kept open for several nights and days, and the manifestations not being checked, but on the contrary, rather promoted by the preachers, who availed themselves of such an opportunity to convert sinners, the emotion extended itself rapidly. The attacks experienced are described by Mr. Cornish as of a nature, in the first instance, resembling attacks of chorea, which afterwards would assume either the character of hysterical or epileptical attacks, and continue, in some instances, for not less than seventy or eighty hours. Children from six years of age to old men of eighty were thus affected, but the cases were mostly of girls and young women. They were chiefly persons of the lowest class and in deplorable ignorance. Mr. Cornish estimates the number of persons affected as not being fewer than four thousand. He was not acquainted with a single case in which it proved fatal." 107.

We all recollect, for it was but yesterday it happened, the exhibition of the gift of tongues among the Irvingites. A startling piece of fanatical enthusiasm in the heart of London, in the nineteenth century. And has not Dr. Elliotson his petites matinées, his charming little soirées of Mesmerism still? If the Okeys have disappeared, the youth Alexis is just come—the somnambulists have been changed, but somnambulism lives—and a pass or two enables the acomplished artiste to read with his rump, and divine and prophesy. All this comes off in an elegant mansion in Conduit-street, in drawing-rooms adorned by modern taste and rich with the treasures of ancient art—the audience the haughty and enlightened aristocracy of free and liberal England—the host and the patron a hospital physician, a lecturer on physic, a physiological writer, a man, who, from every circumstance around him, should have a good right to be, and to be esteemed, philosophical.

" 'Tis true, 'tis pity—pity 'tis, 'tis true."

After all, we of the profession must not bear too hard on peoples' imagination. It does us often a good turn, if, sometimes, it serves us a bad one. How many of the patients of physicians (they have more of this class than the surgeons) are dyspeptic and hypochondriacal from the influence of depressing circumstances, rather than from bodily disorder. How many, in fact, fancy themselves ill, without being so. And what a magical effect confidence *to* them and *in* them has. Tell such with a magisterial air "to take up their bed and walk," and they do so. A joke, a bold dictum, a cynical remark, or a witty one, anything which shows a contempt of the complaint on the doctor's part, and a ready assurance that he can do what he likes with it, lays hold of the fancy of the patient, almost always benefits, and sometimes straightway cures him. This is one of the advantages of celebrity. The doctor, in vogue, effects with a nod, what the poor devil who is not in vogue cannot accomplish at all.

What malleable materials for those who quack, whether in the Faculty or out of it, the bulk of mankind are made of, may be guessed from a fact or two, which we quote at random. We scarcely take a step in practice without stumbling on some of the same description.

"As soon as the powers of nitrous oxide were discovered, Dr. Beddoes at once concluded that it must necessarily be a specific for paralysis: a patient was selected for the trial, and the management of it was entrusted to Sir Humphry Davy. Previous to the administration of the gas, he inserted a small pocket thermometer under the tongue of the patient, as he was accustomed to do upon such occasions, to ascertain the degree of animal temperature, with a view to future comparison. The paralytic man, wholly ignorant of the nature of the process to which he was to submit, but deeply impressed, from the representation of Dr. Beddoes, with the certainty of its success, no sooner felt the thermometer under his tongue than he concluded the *talisman* was in full operation, and in a burst of enthusiasm declared that he already experienced the effect of its benign influence throughout his whole body: the opportunity was too tempting to be lost; Davy cast an intelligent glance at Coleridge, and desired his patient to renew his visit on the following day, when the same ceremony was performed, and repeated every succeeding day for a fortnight, the patient gradually improving during that period, when he was dismissed as cured, no other application having been used." 112.

Professor Woodhouse made some experiments in connection with this same nitrous oxide more satisfactory still, because upon a larger scale, and one, therefore, less open to fallacy.

"At the time that nitrous oxide excited almost universal attention, several persons were exceedingly anxious to breathe the gas; and the professor administered to them ten gallons of atmospherical air, in doses of from four to six quarts. Impressed with the idea that they were inhaling the nitrous oxide, quickness of the pulse, dizziness, vertigo, tinnitus aurium, difficulty of breathing, anxiety about the breast, a sensation similar to that of swinging, faintness, weakness of the knees, and nausea which lasted from six to eight hours were produced." 112.

The religious chapter in the human mind is as mysterious as sacred. The purest of religions consecrates faith, and urges its necessity. The veriest of impostures claims the same, and it is on faith that superstitions have been built, idolatries erected, and miracles performed. The existence of the latter, at all events, their narration, furnish little to be depended on, as intrinsic evidence of truth or falsehood. The cures of Hoenlohë, in our own day, are numerous, well attested, and staggering to those who are not possessed of philosophic minds and medical experience. The cases were of a nervous character; palsy, lameness, defect of sight, of hearing, and so forth. "Dr. Pfeuffer, the directing physician of the Universal Hospital of Bamberg, in his Psychological and Medical Researches respecting these cases, asserts that they were all chronic disorders—not one of an acute character. The cures were undertaken without ostentation or mystery, nor was there any particular manipulation exercised. The zeal and energy, and self-confidence of the prince increased with the various cases that pressed upon him, and the crowd of applicants participated with him in the feeling and excitement." The facts are unquestionable, the explanation not difficult, but the *precise* rationale only to be comprehended when we comprehend, too, the *nature* of the nervous force.

"In the Journal of George Fox, a case of lameness suddenly relieved by an unexpected address under a state of religious ecstasy, is thus recorded: 'After some time I went to a meeting at Arn-side, where Richard Myer was. Now he had been long lame of one of his arms; and I was moved of the Lord to say unto him amongst all the people, Prophet Myer, stand up upon thy legs, (for he was sitting down,) and he stood up, and stretched out his arm, that had been lame a long time, and said, 'Be it known unto you, all people, that this day I am healed.' But his parents could hardly believe it; but after the meeting was done, had him aside, and took off his doublet: and then they saw it was true. He came soon after to Swarth-more meeting, and there declared how that the Lord had healed him. Yet after this the Lord commanded him to go to York with a message from him; and he disobeyed the Lord; and the Lord struck him again, so that he died about three quarters of a year after." 116.

Can we wonder that such cases should influence men, unused to reasoning, excited by enthusiasm, taught that faith is the one great merit, and struck, in their ignorance, by the similarity of the miracle to those which they have revered in Holy Writ. Can *they* nicely balance the weight of evidence, can *they* discriminate the spurious from the true, can *they* distinguish the products of fanaticism from those of inspiration, the first fruits of the Gospel from its remote and incidental grafts, can *they*, enthusiasts, sit in judgment on enthusiasm? When they can, we may expect sectarianism to cease.

The Royal Gift of Healing, religious in its origin, political in its progress, and a farce in its conclusion, demands and receives the most loyal consideration from our author. The antagonism of England and France has appeared in this, as in all things else. Laurentius, physician to Henri Quatre, denied, as in duty bound, that the power resided in the Kings of Britain. The Rev. Dr. Tooker, chaplain to Elizabeth, proved most satisfactorily that it did not belong to the Kings of France. Probably in this, as in many disputes, both parties were right. But this regal prerogative degenerated sadly, for it fell, about the middle of the seventeenth century, into the plebeian hands of a Mr. Valentine Greatrakes; so, at least Mr. Pettigrew spells this gentleman's name, but we apprehend that, from the miracles he did, his unquestionable cures of King's evil, agues, epilepsy, palsy, and convulsions, *Greatshakes* was his real designation. And yet the thing itself was no great shakes, after all, for he made it a present, perhaps in part payment of his wages, to *Mr. John Leverett*, his gardener, who belonging, no doubt, to the Hare family, and being naturally delicate, protested that, "after touching thirty or forty a day, he felt so much goodness go out of him, he was as fatigued as if he had been digging eight roods of ground." A memorable fact, and one that goes a very good way towards confirming the reality of the operations of the Mesmerists, these latter apostles of the touch-and-go system, professing that they feel just the same thing.

We could not conscientiously close this article, intended, in our humble way, to magnify the marvellous, and to discourage that cold scepticism so marring to the poetry of this rail-road age, without some allusion to the sympathetic cures which have immortalised the name of Sir Kenelm Digby, and are, perhaps, too lightly thought of now. For what can be attested better, what more circumstantial, what more historical, and what more satisfactory, than the following case.

"Mr. James Howel, a gentleman celebrated by his 'Dendrologia' and other works, in endeavouring to part two of his friends in a duel, received a severe wound of his hand. Alarmed at this accident, one of the combatants bound up the cut with his garter, took him home, and sent for assistance. The king, upon hearing of the event, sent one of his own surgeons to attend him; but as in the course of four or five days the wound was not recovering very favorably, he made application to Sir Kenelm Digby, of whose knowledge regarding some extraordinary remedies for the healing of wounds he had become apprized. Sir Kenelm first inquired whether he possessed anything that had the blood upon it, upon which Mr. Howel immediately named the garter with which his hand had been bound, which was accordingly sent for. A basin of water being brought, Sir Kenelm put into it a handful of powder of vitriol, and dissolved it therein. He then took the bloody garter, and immersed it in the fluid, while Mr. H. stood conversing with a gentleman in a corner of the room; but he suddenly started, and, upon being asked the reason, replied that he had lost all pain—that a pleasing kind of freshness, as it were a wet cóld napkin had passed over his hand, and that the inflammation, which before had been so tormenting, had vanished. He was then advised to lay aside all his plasters, to keep the wound clean, and in a moderate temperature. After dinner, the garter was taken out of the basin and placed to dry before a large fire; but no sooner was this done than Mr. H.'s servant came running to Sir Kenelm to say that her master's hand had again inflamed, and that it was as bad as before; whereupon the garter was again placed into the liquid, and before the return of the servant all was well and easy again. In the course of five or six days the wound was cicatrized, and a cure performed." 158.

If any are disposed to use that *reason*, so much abused, and to argue that the fact may be explained, consistently with our knowledge of the nature of wounds, the right mode of dressing them, and a liberal allowance for the credulousness of the actors, and for a circumstance generally admitted to be true, that a good story never loses in the telling, we bid them hold their peace and go their way, and not intrude their wretched philosophy upon us.

We have done. The superstitions connected with medicine have been many—the humbug still mixed up with it is great—it has jumped with our humour to laugh at the one, and possibly to indulge in a sneer at the other—they have both amused us in an idle hour—and we only hope that a few poor jokes, and a little raillery may be taken in good part by those who have leisure to glance at such a trifle.

